# Anthelmintic resistance in livestock in Africa: review of the current status

**DOI:** 10.1186/s12917-025-05077-0

**Published:** 2025-10-17

**Authors:** Lucy Gatitu, Mitchelle R. Kasudi, Samuel Githigia, Arshnee Moodley, Dishon M. Muloi

**Affiliations:** 1 Health Program, International Livestock Research Institute, Nairobi, Kenya; 2 Department of Veterinary Pathology, Microbiology and Parasitology, University of Nairobi, Nairobi, Kenya; 3 Department of Veterinary and Animal Sciences, University of Copenhagen, Frederiksberg C, Denmark; 4 Institute of Infection, Veterinary and Ecological Sciences, University of Liverpool, Neston, UK

**Keywords:** Gastrointestinal nematode, Anthelmintic resistance, Livestock, Africa

## Abstract

**Background:**

Livestock across the globe are frequently infected with multiple gastrointestinal nematode (GIN) species, and the use of anthelmintics remains a cornerstone of their control. However, anthelmintic resistance (AHR) poses a growing threat to sustainable livestock production, resulting in reduced productivity and compromised animal health and welfare. Reports of resistance in various helminth species against multiple anthelmintic classes exist in African livestock; however, studies summarising the extent and burden of this resistance are limited, and systematic monitoring or surveillance efforts are largely absent across the continent.

**Methods:**

We conducted a comprehensive scoping review to evaluate the current status of AHR in African livestock.

**Results:**

Our systematic search yielded 357 original studies, 28 met the eligibility criteria, covering nine countries and spanning from 1996 to 2024. All studies involved cattle and/or small ruminants, focusing primarily on two anthelmintic classes—benzimidazoles and macrocyclic lactones—and two gastrointestinal nematode genera: *Haemonchus* and *Trichostrongylus*. Resistance was reported across most of the studies, although reported prevalence rates were highly heterogeneous, varying considerably with anthelmintic class, livestock species, and geographic location. We observed variability in study methodologies, including differences in faecal egg count reduction test (FECRT) design, sampling intervals, dosage regimens, and reporting quality, which potentially limits comparability across countries and restricts epidemiological insight.

**Conclusions:**

These findings highlight the urgent need to more accurately quantify the burden of AHR and to establish coordinated surveillance systems across Africa. Equally important is the development of sustainable parasite control strategies and the promotion of responsible anthelmintic use to preserve the efficacy of existing drugs.

**Supplementary Information:**

The online version contains supplementary material available at 10.1186/s12917-025-05077-0.

## Background

Livestock production significantly contributes to household income in Africa, with over 70% of households relying on it for up to 33.8% of their total income [1]. However, infections with helminth parasites, particularly gastrointestinal nematodes (GIN), pose a major threat to the profitability and sustainability of livestock systems. These infections are typically chronic and lead to subclinical losses, including reduced weight gain, lower meat and milk yields, diminished reproductive performance, and overall compromised animal health and welfare [2–4]. A recent meta-analysis reported that GIN infections in sheep resulted in reductions of 58% in weight gain, 90% in wool production, and 78% in milk yield [2]. GIN infections are more prevalent in animals with outdoor access, such as cattle and small ruminants [5], but also found in indoor reared species such as pigs and poultry.

Current control strategies rely heavily on the use of broad-spectrum anthelmintics, primarily benzimidazoles (e.g., albendazole), imidazothiazoles (e.g., levamisole), and macrocyclic lactones (e.g., ivermectin), administered either curatively or prophylactically [6]. However, the frequent administration of anthelmintics, often with incorrect dosing or without rotation of different drug classes, has led to the development of anthelmintic resistance (AHR).

The global and national burden of GIN infections and AHR remains unknown, but available data suggest that helminth infections cost the European livestock industry over $2 billion annually, with AHR alone accounting for an estimated $41 million in losses [7]. These economic costs are expected to rise due to the spread of resistant parasites, including multi-drug resistant strains. Additionally, climate change is likely to exacerbate parasite transmission, leading to more frequent treatments and further resistance development [8]. In much of Africa, where helminths have long seasonal activity, anthelmintic drugs are widely accessible and commonly used, often without veterinary guidance, resulting in rapid acceleration of AHR. Several studies have reported the occurrence of AHR in multiple helminth species against different anthelmintic classes in different livestock species [9]. Effective resistance management requires the ability to detect its presence and support drug-use decisions regarding effective treatments against target helminth populations. Routine anthelmintic efficacy testing in most countries is undertaken through the faecal egg count reduction test (FECRT) [10], which remains the gold standard recommended by the World Association for the Advancement of Veterinary Parasitology (WAAVP) [11]. Data is essential to measure the extent of this problem in Africa. However, robust data to assess the scale of AHR across the continent are scarce, and systematic monitoring or surveillance frameworks are currently lacking.

This scoping review aimed to comprehensively map the scientific evidence reporting anthelmintic resistance in GINs in different livestock species in Africa. Our findings will support anthelmintic stewardship and development of effective parasite control strategies; and underscore the need for investment in routine surveillance systems and further research.

## Materials and methods

### Data sources, search strategy and screening

We conducted a scoping review to collect evidence on the prevalence of AHR in livestock across African countries. The review followed the Preferred Reporting Items for Systematic Reviews and Meta-Analyses extension for Scoping Reviews (PRISMA-ScR) guidelines [12] (Supplemental material 2). A comprehensive search was conducted in three electronic databases – PubMed (MEDLINE), Web of Science, and CABI – using a combination of search terms related to anthelmintic resistance (“anthelmintic resistance” OR “endoparasite resistance” OR “antiparasitic resistance” OR “nematode resistance” OR “benzimidazole resistance” OR “imidazothiazole resistance” OR “salicylanilide resistance” OR “tetrahydropyrimidine resistance” OR “macrocyclic lactones resistance”), food animals (cattle OR bovine OR sheep OR ovine OR goat OR caprine OR chicken OR poultry), and African countries. No date restrictions were applied, and the search included studies published up to 3 February 2025. A detailed search strategy for each database is provided in the Supplemental material 1. Studies were eligible for inclusion if they were peer-reviewed, original research articles reporting the prevalence of AHR using FECRT method in sheep, goats, cattle, pigs, or poultry in African countries. Case reports, review articles, and studies available in non-English full texts were excluded. Titles and abstracts retrieved from the database searches were screened for eligibility. Full texts of potentially eligible articles were then reviewed in detail to confirm inclusion. Data extraction was performed using a standardised template. Initial screening and data extraction were conducted by one author (LG), with independent verification by a second author (DMM or MRK).

## Data extraction and synthesis of results

One author (LG) extracted data from the full texts of eligible studies using a piloted, standardised data collection form. A second reviewer (MRK or DMM) independently verified all extracted information to ensure accuracy. Extracted data included: article title, first author, year of study and publication, study design, country of study, number and age of animals treated, specific anthelmintic(s) or combinations used, diagnostic tests employed, route of drug administration (oral or injectable), dosage, and anthelmintic efficacy where available. Study quality was assessed using an adapted version of the Joanna Briggs Institute critical appraisal tool for prevalence studies. A score of 1 was assigned for each criterion met, 0.5 if partially met, and 0 if not met. Scores were calculated based on the responses to each question [13]. Appraisal scores were categorised as very low (≤ 25%), low (26–50%), moderate (51–75%), or high (> 75%).

In line with established scoping review methodology, we used tabular and thematic approaches to summarise the extracted data. Descriptive statistics, including counts and proportions, were used to characterise the included studies. Anthelmintic efficacy was assessed using the faecal egg count reduction test (FECRT) where drug efficacy ≥ 95% indicates susceptibility in the parasite population, while efficacy < 95% suggests resistance.

## Results

### Characteristics of included studies

**Fig. 1:**
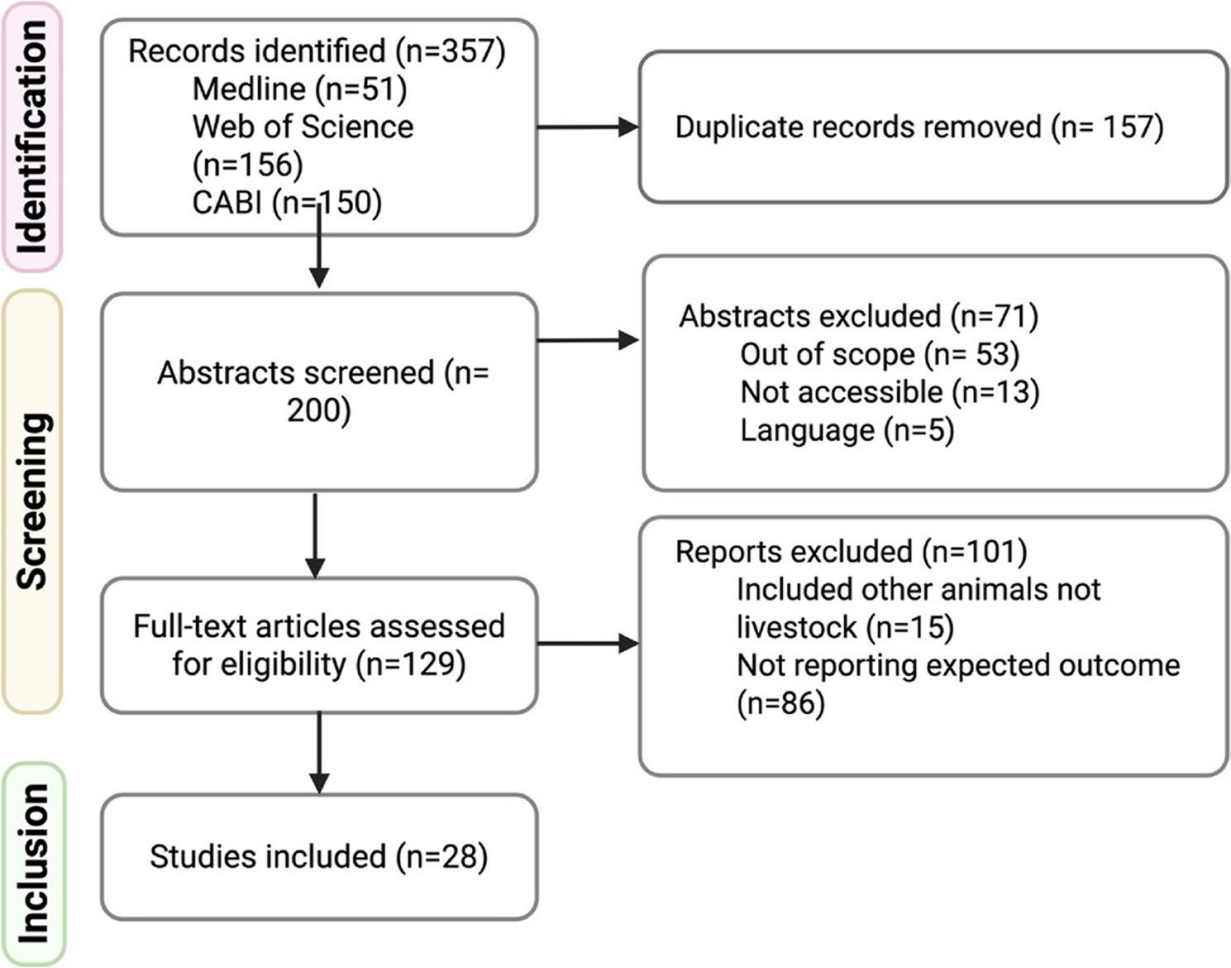
Study selection flowchart

A total of 357 studies were identified through the database search. After removing duplicates, 200 articles were screened by title and abstract, of which 129 were selected for full-text review. Ultimately, 28 articles met the inclusion criteria and were included in this scoping review (Fig. 1). The characteristics of each include study are detailed in Table 1.

**Table 1:** Description of selected studies

Author	Country	Livestock species	Number of animals	Anthelmintic Tested	Dosage	Route of administration	Efficacy < 95%	FECRT Design and post-treatment sampling interval	Helminth genus
Bentounsi, et al. 2012 [14]	Algeria	Cattle	186	Albendazole	7.5 mg/kg	Oral	58%−83%	Unpaired Day 0–20	Trichostrongylus
Atanasio, et al. 2002 [15]	Mozambique	Goats	568	Albendazole	5 mg/kg	Oral	43–82.9.9%	Unpaired Day 0–10	Haemonchus Trichostrongylus Oesophagostomum
				Fenbendazole	5 mg/kg	Oral	49–87.6.6%		
				Levamisole	7.5 mg/kg	Oral	87.4–93.5%		
Bersissa and Girma, 2009 [16]	Ethiopia	Goats	200	Albendazole	7.5 mg/kg	Oral	48–67%	Unpaired Day 0–12	Haemonchus Trichostrongylus Teladorsagia
				Tetramisole	22.5 mg/kg	Oral	80–84%		
				Ivermectin	0.2 mg/kg	SC	71–78%		
Waruiru et al. 1998 [17]	Kenya	Goats	1300	Albendazole	5 mg/kg	Oral	29.0%	Unpaired Day 0–14	Haemonchus Oesophagostomum Trichostrongylus
				Thiophanate	50 mg/kg	Oral	16.9%		
				Levamisole	15 mg/kg	Oral	62.9%		
				Rafoxanide	7.5 mg/kg	Oral	79.2%		
				Ivermectin	0.2 mg/kg	Oral	S(99.8%)		
				Ivermectin	0.2 mg/kg	SC	S(99.2%)		
Getachew, et al. 2016 [18]	Ethiopia	Sheep	141	Albendazole	7.5 mg/kg	Oral	57.5–92%	Unpaired Day 0–14	Strongyles Trichuris
				Tetramisole	15 mg/kg	Oral	S (98.6–100)		
				Ivermectin	0.2 mg/kg	SC	51.7–87.6%		
Boersema and Pandey 1997 [19]	Zimbabwe	Sheep	40	Fenbendazole	5 mg/kg	Oral	0–80%	Unpaired Day 0–14	Trichostrongylus Haemonchus Oesophagostomum
				Levamisole	5 mg/kg	SC	46–91%		
				Rafoxanide	7.5 mg/kg	Oral	32–79%		
Mohammedsalih et al. 2020 [20]	Sudan	Goats	60	Albendazole	Unpaired FECRT 5 mg/g	Oral	85.8–87.5%	Paired and unpaired Day 0–14	Haemonchus Trichostrongylus Cooperia
					7.5 mg/kg		87.1%		
					10 mg/kg		86.5–87.3%		
					12.5 mg/kg		96.6%		
					Paired FECRT 5 mg/kg		54.4–90.2%		
					7.5 mg/kg		93.7%		
					10 mg/kg		87.3–89.8%		
					12.5 mg/kg		97.2%		
Wondimu and Bayu 2022 [21]	Ethiopia	Goats	100	Albendazole	7.5 mg/kg	Oral	69.9%	Unpaired Day 0–14	Trichostrongylus Haemonchus Teladorsagia
				Tetraclozan	7.5 mg/kg	Oral	84.3%		
				Tetramisole,	7.5 mg/kg	Oral	S (95.7) %		
				Ivermectin	0.2 mg/kg	SC	71.1%		
Mphahlele, et al. 2021 [22]	South Africa	Sheep	200	Albendazole	7.5 mg/kg	Oral	47–92%	Unpaired Day 0–14	Haemonchus Trichostrongylus Teladorsagia
				Levamisole	5 mg/kg	Oral	70–94%		
				Ivermectin	0.2 mg/kg	SC	65–92%		
Bakunzi et al. 2008 [23]	South Africa	Sheep	480	Albendazole	7.5 mg/kg	Oral	68–93%	Unpaired Day 0–14	Haemonchus Oesophagostomum Trichostrongylus
				Levamisole	7.5 mg/kg	Oral	58–91%		
				Closantel	5 mg/kg	Oral	72–94%		
Nsereko, et al. 2013 [24]	Uganda	Goats	93	Albendazole	5 mg/kg	Oral	77.30%	Unpaired Day 0–14	Haemonchus Oesophagostomum
				Levamisole	7.5 mg/kg	Oral	85%		
				Ivermectin	0.2 mg/kg	SC	54–92%		
Byaruhanga et al. 2013 [25]	Uganda	Goats	71	Albendazole	5 mg/kg	Oral	11–28.5.5%	Unpaired Day 0–13	Haemonchus Cooperia Oesophagostomum
				Levamisole	7.5 mg/kg	Oral	84.8–91%		
				Ivermectin	0.2 mg/kg	SC	78.47%		
Gabriel et al. 2001 [26]	Zambia	Sheep	360	Albendazole	4 mg/kg	Oral	48–86%	Unpaired Day 0–14	Haemonchus
				Levamisole	7.5 mg/kg	Oral	S(95–99.4.4%)		
				Ivermectin	0.2–0.3 mg/kg	Oral	72–94%		
Chaka and Gizaw 2009[27]	Ethiopia	Sheep	120	Albendazole	7.5 mg/kg	ND	30.4%	Paired Day 0–21 for ivermectin 0–15 for albendazole and tetramisole	Haemonchus
				Tetramisole	15 mg/kg		75%		
				Ivermectin	0.3 mg/kg		78.5%		
Dreyer 2002 [28]	South Africa	Sheep	50	Albendazole	3.8 mg/kg	Oral	6.3–86.9%	Unpaired Day 0–10	ND
				Levamisole	7.5 mg/kg	Oral	7.9–87%		
				Ivermectin	0.2 mg/kg	Oral	76.2–87%		
				Moxidectin	0.2 mg/kg	Oral	94.2%		
Mohammedsalih et al. 2021 [29]	Sudan	Cattle	123	Albendazole	7.5 mg/kg	Oral	Unpaired: 88.2%−93.7%	Paired and unpaired 0-day 14	Haemonchus Trichostrongylus Oesophagostomum Cherbatia
							Paired: 88.5–94.6%		
Nabukenya, et al. 2014 [30]	Uganda	Goats	497	Albendazole	7.5 mg/kg	Oral	40–94%	Unpaired Day 0–15	Haemonchus Strongyloides Trichostrongylus Cooperia Bunostomum
				Levamisole	10.5 mg/kg	Oral	29–92%		
				Ivermectin	0.3 mg/kg	SC	54–92%		
Mukaratirwa et al. 1997[31]	Zimbabwe	Sheep	226	Albendazole	10 mg/kg	Oral	56–87%	Paired Day 0–7	Haemonchus Cooperia
				Oxfenbendazole	5 mg/kg	Oral	82.3–82.9%		
				Fenbendazole	5 mg/kg	Oral	83.3%		
				Levamisole	7.5 mg/kg	Oral	89.9%		
				Ivermectin	0.02 mg/kg	SC	S (100%)		
Tsotetsi, et al. 2013[32]	South Africa	Sheep	120	Albendazole	7.5 mg/kg	Oral	56–76%	Paired Day 0–14	Haemonchus Trichostrongylus Teladorsagia
				Levamisole	5 mg/kg	Oral	81–87%		
				Ivermectin	0.2 mg/kg	SC	76–87%		
		Goats	80	Albendazole	7.5 mg/kg	Oral	22–77%		
				Levamisole	5 mg/kg	Oral	60–92%		
				Ivermectin	0.2 mg/kg	SC	I: 27%		
Bentounsi et al. 2007 [33]	Algeria	Sheep	192	Albendazole	3.8 mg/kg	Oral	21–94%	Unpaired FECRT Day 0–10	Teladorsagia Trichostrongylus Marshallagia Nematodirus
				Ivermectin	0.0002 mg/kg	SC	24–89%		
Molla, et al. 2023 [34]	Ethiopia	Sheep	735	Albendazole	ND	ND	68–93.5.5%	Unpaired FECRT Day 0–14	Strongylid Fasciola
				Triclabendazole					
Bakunzi 2003 [35]	South Africa	Goats	400	Fenbendazole	5 mg/kg	Oral	47–88%	Unpaired FECRT Day 0–14	Haemonchus Trichostrongylus Oesophagostomum
				Levamisole	7.5 mg/kg	Oral	76–93%		
				Rafoxanide	7.5 mg/kg	Oral	31–91%		
Wanyangu, et al. 1996 [36]	Kenya	Sheep	1328	Albendazole Levamisole	ND	ND	ND	Unpaired FECRT Day 0–14	Haemonchus Trichostrongylus Oesophagostomum
		Goats	989	Albendazole Levamisole					
Maingi, et al. 1998 [37]	Kenya	Sheep	ND	Albendazole	5 mg/kg	Oral	ND	Unpaired FECRT Day 0–14	Haemonchus
				Levamisole	7.5 mg/kg	Oral			
				Febantel	5 mg/kg	Oral			
Bentounsi, et al. 2006 [38]	Algeria	Sheep	29	Albendazole	ND	ND	71%	Unpaired FECRT Day 0–10	Strongles
				Fenbendazole					
							77%		
Atanásio-Nhacumbe, et al. 2017 [39]	Mozambique	Goats	335	Albendazole	5 mg/kg	Oral	0–81%	Unpaired FECRT Day 0–14	Haemonchus Oesophagostomum Trichostrongylus Strongyloides
Emsley et al. 2023 [40]	South Africa	Sheep Goats	130 119	Albendazole	7.5 mg/kg	Oral	3.81–72.15%	FECRT Day 0–14	Haemonchus
				Levamisole	5 mg/kg	Oral	10–72.95.95%		
				Ivermectin	0.2 mg/kg	SC	43.66–54.66%		
Maurizio, et al. 2024 [41]	Ethiopia	Cattle	120	Albendazole	ND	Oral	52.2–68.1%	Unpaired FECRT Day 0–14	ND
				Tetramisole		Oral	94.2%		
				Ivermectin		SC	56.5–73.8%		
		Goats	120	Albendazole		Oral	82.5–91.5%		
				Tetramisole		Oral	S		
				Ivermectin		SC	88.8–94.8%		
		Sheep	120	Albendazole		Oral	85.1–91.1%		
				Tetramisole		Oral	92.1%		
				Ivermectin		SC	93.4–94.1%		

*ND Not defined, *S* Susceptible, *SC* subcutaneous

**Fig. 2:**
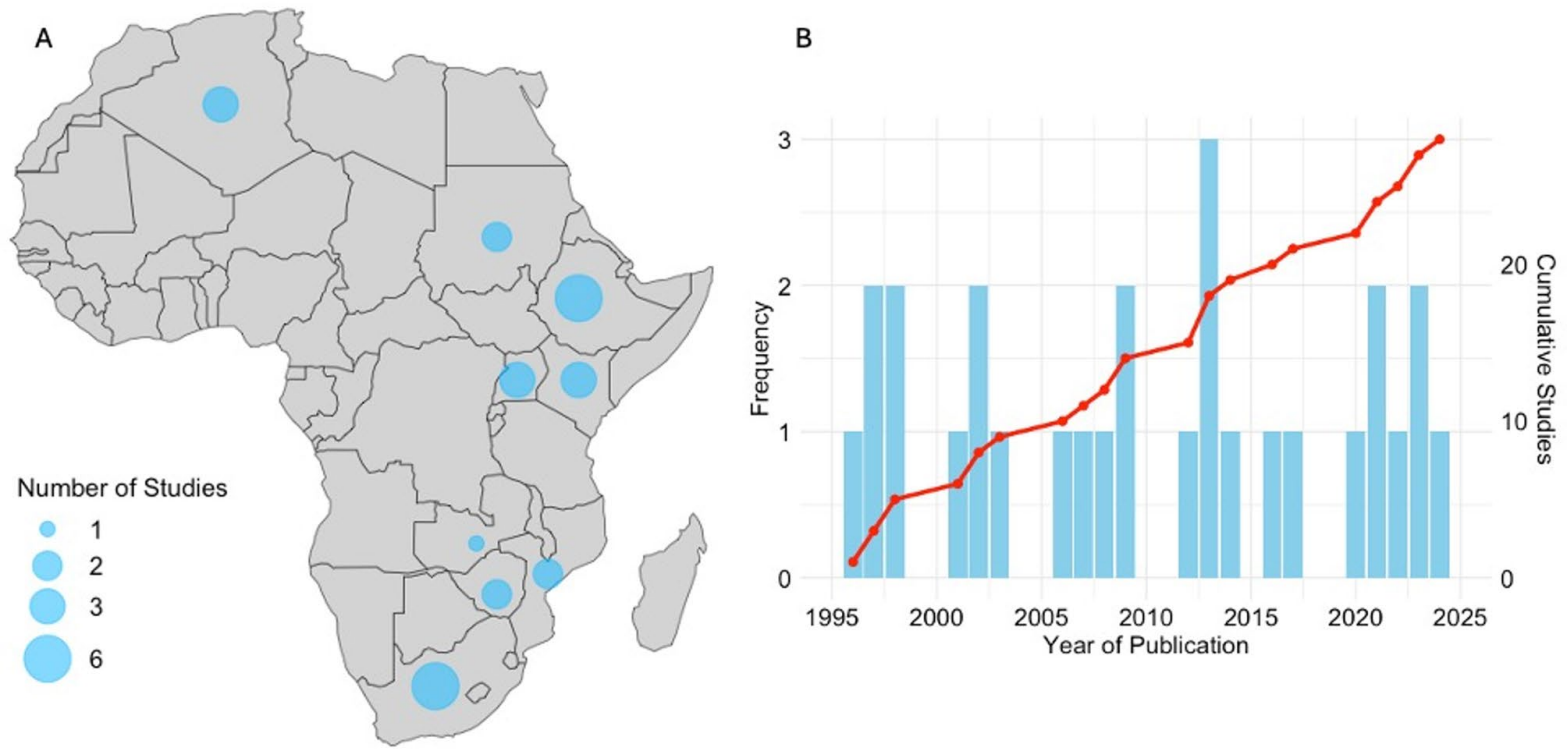
**A**-**B**. **A** Regional distribution of included studies. **B** Number of studies published over time. The left y-axis shows the annual number of papers, while the right y-axis and trend line represent the cumulative total of studies

The included studies originated from nine African countries—representing 16.7% of the continent’s 54 nations—indicating limited geographical coverage. South Africa and Ethiopia each contributed six studies, followed by Uganda, Algeria, and Kenya with three studies each. Zimbabwe, Sudan, and Mozambique contributed two studies each, and Zambia contributed one (Fig. 2A). The selected articles were published between 1996 and 2024, with no clear linear publication trend. Notably, 68% (*n* = 19) of the included studies were published before 2014 (Fig. 2B).

Among the 28 studies included, 12 (42.9%) investigated AHR exclusively in sheep, 10 (35.7%) in goats, and two (7.1%) in cattle. Three studies (10.7%) included both sheep and goats, while one (3.6%) assessed all three ruminant species. No studies evaluated AHR in non-ruminant livestock such as pigs or poultry. Resistance was investigated against five main anthelmintic classes either individually or in combination: benzimidazoles (albendazole, fenbendazole, oxfenbendazole, thiophanate, triclabendazole, febantel), imidazothiazoles (levamisole, tetramisole), salicylanilides (closantel, rafoxanide, tetraclozan), macrocyclic lactones (ivermectin, moxidectin, doramectin), and tetrahydropyrimidines (morantel). Benzimidazoles were the commonly studied drug, reported in all 28 studies either singly or in combination, followed by imidazothiazoles in 21 studies (75%) and macrocyclic lactones in 16 studies (57.1%). Salicylanilides were evaluated in five studies (17.9%). A total of 10 different parasite genera were reported across the 28 studies. *Haemonchus spp* was the most frequently reported parasite, investigated in 21 studies (75%). *Trichostrongylus* spp. were reported in 16 studies (57.1%), *Oesophagostomum* spp. in 10 (35.7%), *Teladorsagia* spp. In 6 (21.4%), and *Cooperia* spp. in 4 (14.3%). Less frequently reported were *Strongyloides* (2 studies (7.1%), *Bunostomum*, *Trichuris*, Cherbatia, *Marshallagia*, and *Nematodirus* (each in 1 study, 3.6%).

### Methodological quality assessment

Across the 28 studies, the majority (*n* = 22, 78.6%) employed an unpaired FECRT design, in which you compare treated animals to untreated controls. Three studies (10.7%) used a paired design [27, 31, 32], following the same animals before and after treatment, while two studies (7.1%) applied both approaches [20, 29]. In one of the studies using both approaches, paired approach yielded lower efficacy estimates compared to unpaired approach [20] (Unpaired: 85.8–87.5% vs. paired 54.4–90.2% for 5% albendazole) while in the second study both approaches showed close agreement (Unpaired: 88.2%−93.7% vs. Paired: 88.5–94.6% for 7.5% albendazole) [29]. Post-treatment sampling intervals were generally consistent with WAAVP recommendations. Most studies collected samples on day 14 (*n* = 18, 64.3%), followed by day 10 (*n* = 4, 14.3%), while day 7, day 12, day 13, day 20 and day 21 were each reported in a single study (3.6% each), and two studies reporting day 15 (3.6%). Sample sizes varied considerably across studies, ranging from 29 to 1,328 animals. Most studies (57.1%, *n* = 18) enrolled between 100 and 500 animals, while 21.4% (*n* = 6) included fewer than 100 animals and 14.3% (*n* = 5) had more than 500. Larger sample sizes were mostly reported in sheep and goat studies, whereas cattle studies were fewer and typically involved smaller cohorts of around 120–186 animals. Dosage regimens varied both across drug classes and even within the same host species. Albendazole showed the widest range, administered at doses ranging from 3.8 mg/kg to 12.5 mg/kg, with one study using 3.8 mg/kg, eight studies using 5 mg/kg, seven studies using 7.5 mg/kg, two studies using 10 mg/kg, and one study testing 12.5 mg/kg.

Nearly one-third of studies (*n* = 10, 35.7%) were rated as low quality, 11 (39.3%) were of moderate quality, and seven (25%) achieved a high-quality rating (Supplemental Table S1). When assessed by quality domain, reporting was strongest in the description of the anthelmintic intervention including drug, dosage, and route of administration, as well as in the method of parasite identification and quantification (each mean score 0.9). The FECRT procedure and efficacy threshold were also generally well described (mean score 0.83). In contrast, reporting of the study design (mean score 0.70) and the target animal species and sourcing (mean score 0.69) was less consistent. Only six studies clearly differentiated helminth egg species in their FECR reporting (mean score 0.22), and just one study accounted for sample size or incorporated a formal power calculation.

### Resistance to benzimidazoles

Of the 28 studies reporting benzimidazole resistance, 26 (93.9%) documented resistance to albendazole, five (17.9%) to fenbendazole, and one each (3.6%) to oxfenbendazole and thiophanate. Efficacy within this class was highly variable, with albendazole showing the widest range. Nearly half of the studies (42.9%, *n* = 12) on albendazole reported efficacy below 50%, including complete treatment failure in goats in Mozambique (0% efficacy) [39], 11–28.5.5% in goats in Uganda [25], 29% in goats in Kenya [17], and 3.8–72.1% in South African small ruminants [40]. In contrast, other studies demonstrated higher efficacy. Fenbendazole efficacy also varied widely, with 40% (*n* = 2) of studies reporting reductions below 50% (0–80% in sheep in Zimbabwe [19], 47–88% in goats in South Africa [35]).

### Resistance to imidazothiazoles

Among the 21 studies that assessed resistance to imidazothiazoles, 16 (81.3%) evaluated levamisole and five (23.8%) tetramisole. Levamisole generally showed better efficacy, with most studies reporting rates above 80%. For example, efficacy reached 85% in goats in Uganda [24, 25], 89.9% in sheep in Zimbabwe [31], 76–93% in sheep and goats in South Africa [35] and goats in Mozambique [15]. However, approximately one-third of the studies (30.7%, *n* = 4) documented efficacy values below 50%, with the lowest reported at 7.9% in sheep in South Africa [28]. Tetramisole showed generally higher efficacy, with all studies reporting rates greater than 75%.

### Resistance to salicylanilides

Five studies assessed resistance to salicylanilides, including three on rafoxanide, one on closantel, and one on tetraclozan. Rafoxanide showed high efficacy in most studies; however, two reported lower efficacy rates, with values as low as 31% [35] in goats in South Africa and 32% in sheep in Zimbabwe [19]. Closantel demonstrated consistently high efficacy (72–94%) in South African sheep [23], while tetraclozan achieved 84.3% in goats in Ethiopia [21].

### Resistance to macrocyclic lactones

Resistance to ivermectin was reported in 16 studies (57.1%), with efficacy ranging widely. Two studies documented efficacy below 50%, including 24% in Algeria [33], 27% in goats in South Africa [32],and 43% in sheep in South Africa [40]. Moderate reductions were reported in Uganda (54–92%) [30] and Ethiopia (51.7–87.6%) [18], while higher efficacy was observed in Zambia (72–94%) [42] and Kenya, where both oral and subcutaneous dosing achieved >99% [17]. A multi-host study from Ethiopia highlighted variability by species, with efficacy lowest in cattle (56.5–73.8%) compared to goats (88.8–94.8%) and sheep (93.4–94.1%) [41]. Moxidectin was evaluated in a single study and demonstrated high efficacy of 94.2% [28].

## Discussion

Our findings highlight a striking scarcity of data on AHR in livestock across Africa. Given that the continent is home to more than a quarter of the global livestock population – comprising 20% of the world’s cattle, 27% of sheep, and 32% of goats [43] – and these numbers are rising, the limited number of studies in our review underscores a critical research and surveillance gap. It is indisputable that anthelmintic resistance constitutes a substantial component of the broader antimicrobial resistance (AMR) challenge in livestock [6]. Yet, research and surveillance on anthelmintic resistance remain largely excluded from funding priorities and implementation strategies aimed at understanding and addressing AMR. The disproportionate focus on bacterial AMR in animal health, primarily driven by public health reasons, is concerning, as effective anthelmintic protection is essential not only for animal health but also for food security across the region. There is a pressing need for increased funding to support the development of these systems, strengthen laboratory infrastructure, harmonise field and laboratory standard operating procedures, and improve coordination between animal health policy makers, livestock producers, and users of anthelmintics. We call for renewed national and regional efforts to establish and operationalise robust anthelmintic resistance surveillance systems.

Whilst most studies broadly adhered to WAAVP guidelines on FECRT implementation, methodological inconsistencies limit the comparability and interpretability of results across settings and host species. For example, minimal description of study design such as animal selection or lack of standardisation in dosing regimens even within the same host species. Likewise, limited differentiation of helminth species in FECRT results constrains the ability to link resistance patterns to specific parasites, hindering epidemiological insights needed for targeted control. In 2023, WAAVP issued updated guidelines to improve methodology and standardisation of FECRT in livestock with an aim of supporting researchers, practitioners, and policymakers in the diagnosis and classification of AHR [44]. These guidelines recommend prioritising paired study designs over unpaired ones, basing requirements on the total number of eggs counted rather than mean EPG, and allowing flexible treatment group sizes according to expected egg counts.

Collectively, the studies reviewed demonstrate evidence of resistance to commonly used anthelmintics across multiple livestock species and African countries. Several factors are likely contributing to this observation. First, the frequent and often indiscriminate use of anthelmintics, whether given prophylactically or at low doses due to cost [45] and the heavy reliance on a few drug classes like benzimidazoles (e.g., albendazole), macrocyclic lactones (e.g., ivermectin), and imidazothiazoles (e.g., levamisole). In addition, poor farm management practices, such as the absence of pasture rotation and high stocking densities [46], combined with the lack of systematic monitoring programs for detecting resistance contributes to the continued use of ineffective treatments [47]. The rapid emergence of resistance to new anthelmintic classes, often within a decade of their introduction [48], is a growing concern [49]. With no new broad-spectrum anthelmintics featuring novel mechanisms of action developed since the introduction of macrocyclic lactones in the 1980 s [50], the threat posed by anthelmintic resistance is particularly serious. Climate change is also expected to accelerate the spread and severity of parasitism [8, 51, 52]. Rising temperatures and prolonged transmission seasons will increase parasite burdens and treatment frequency, which, in turn, will hasten resistance development. Understanding the interaction between climate change and AHR is therefore essential to devising long-term, sustainable control strategies [53]. In light of anticipated climate shifts, transitioning toward non-chemical parasite control methods are likely to become increasingly critical. In recent years, considerable effort has gone into quantifying the economic impact of GIN infections and AHR in livestock, but most analyses are limited to high income settings [7]. Quantifying these hidden losses would provide producers actionable information for on-farm decision-making and incentivise the uptake of non-pharmaceutical control measures while giving veterinary authorities the evidence base to advocate—credibly—for investment in surveillance and targeted intervention programmes.

All studies reviewed focused on cattle and/or small ruminants and primarily examined two classes of anthelmintics, benzimidazoles and macrocyclic lactones and two GINs *Haemonchus spp* and *Trichostrongylus spp*. The emphasis on ruminants likely reflects both ecological factors, particularly their high susceptibility to gastrointestinal nematodes due to grazing behaviour, and historical research priorities. Similarly, the predominance of benzimidazoles and macrocyclic lactones corresponds with their widespread use in livestock across sub-Saharan Africa, driven by factors such as affordability, accessibility, and ease of administration [54, 55].The dominance of *Haemonchus* and *Trichostrongylus* in the dataset likely reflects their high pathogenic potential and adaptive capacity, as well as their well-documented resistance profiles [56].These findings should inform the strategic prioritisation of AHR surveillance efforts across the continent, focusing on key parasite genera, anthelmintic classes, and livestock species.

The FECR test was the most widely used method for assessing anthelmintic efficacy in vivo and estimating the prevalence of resistance in our review. The lack of standardized FECR calculation methods across studies—with some comparing treated and control groups, and others focusing on pre- and post-treatment counts—could lead to discrepancies in the interpretation and comparison of resistance levels. According to the most recent WAAVP guidelines, FECR should be calculated using a paired design (pre- and post-treatment faecal egg counts from the same animals) as this approach enhances statistical power, controls for inter-animal variability, and yields more robust estimates of anthelmintic efficacy than post-treatment comparisons between treated and control groups [44]. Further, the FECR test is constrained by low sensitivity, high costs, reliance on laboratory infrastructure, and labour-intensive sampling making it largely inaccessible to veterinarians and farmers across African countries and thus limiting its utility in targeted anthelmintic stewardship. Veterinary authorities should prioritise expanding access (and affordability) to anthelmintic resistance diagnostics across regional laboratories, complementing existing investments in antibiotic resistance surveillance. A key scientific priority remains the development of rapid, reliable tools and protocols for early detection of anthelmintic resistance, to support timely and effective responses.

This scoping review has several limitations. First, our literature search was limited to English-language articles and did not include grey literature or supplementary search strategies such as citation tracking. Consequently, relevant studies may have been omitted. Second, data screening and extraction were initially performed by a single reviewer; although these data were subsequently verified by a second reviewer and discrepancies were resolved through team consensus, inadvertent errors or missed information cannot be completely ruled out. Third, the limited number of identified studies and their narrow geographic coverage - encompassing only nine African countries - should be considered when interpreting our findings. Finally, some of the included studies provided insufficient details regarding data sources or clear descriptions of their methodological approaches, potentially affecting the robustness and interpretability of the synthesized evidence.

## Conclusion, recommendations and implications for decision-making

Our scoping review highlights clear evidence of AHR in livestock across Africa; however, research in this area remains disproportionately limited relative to the scale and significance of the continent’s livestock sector. Several key recommendations arise from our findings. Firstly, increased investment and research from a wider range of African countries is essential to generate comprehensive, geographically representative data on AHR. Currently, the limited available data reflect broader global neglect of AHR within the antimicrobial resistance agenda, which tends to prioritise bacterial pathogens. Yet, the implications of AHR are profound, including diminished animal health and productivity, higher treatment costs, and increased susceptibility to climate-driven parasitic challenges. Secondly, establishing standardised surveillance systems to systematically monitor AHR development and spread should be a high priority for governments. Such surveillance should routinely gather basic clinical, epidemiological, drug-efficacy data, and climatic data where possible. Thirdly, veterinary authorities should advocate policies restricting routine prophylactic anthelmintic use, promoting instead targeted, evidence-based treatment strategies that prioritize high-risk animal populations and geographical hotspots. The development and broader adoption of non-chemical parasite control methods are essential, particularly given increased climate variability and a lack of novel anthelmintic classes. Finally, quantifying the economic impacts of GIN infections and associated AHR in African livestock is critical to guide investment decisions, inform policy development, and support sustainable livestock production and livelihoods. Addressing these gaps will require strengthened collaboration among governments, funding agencies, animal health authorities, researchers, veterinary professionals, and livestock producers.

## Supplementary Information

Supplementary Material 1.

Supplementary Material 2.

## Data Availability

The dataset analysed during the current study is provided in the manuscript text.

## Abbreviations

AHR: Anthelmintic resistance
AMR: Antimicrobial resistance
FECRT: Faecal egg count reduction test
GIN: Gastrointestinal nematode
PRISMA-ScR: Preferred Reporting Items for Systematic Reviews and Meta-Analyses extension for Scoping Reviews
