# Supplementary material for: Anthelmintic resistance in livestock in Africa: review of the current status
Source: BMC Vet Res. 2025 Oct 17;21:615. doi: 10.1186/s12917-025-05077-0 (PMC12534917; doi:10.1186/s12917-025-05077-0)
Supplement: Supplementary file 1 — Supplementary Material 1. [file 12917_2025_5077_MOESM1_ESM.docx]

**Supplemental Material Data**

**Search strategy in three databases**

**CABI DIRECT**

(ab:( ‘**Anthelmintic resistance**’) OR ab:(‘**Endoparasite resistance**’) OR ab:(‘**Antiparasitic resistance**’) OR ab:(‘**nematode resistance**’) OR ab:(‘**Benzimidazole resistance**’) OR ab:(‘**imidazothiazole resistance**’) OR ab:(‘**salicylanilide resistance**’) OR ab:(‘**Tetrahydropyrimidine resistance**’) OR ab:(‘**Macrocyclic lactones resistance’) AND (ab:(‘cattle**’) OR ab:(‘**bovine**’) OR ab:(‘**sheep**’) OR ab:(‘**ovine**’) OR ab:(‘**goat**’) OR ab:(‘**caprine**’) OR ab:(‘**chicken**’) OR ab:(‘**poultry’) AND (ab:(‘Algeria**’) OR ab:(‘**Angola**’) OR ab:(‘**Benin**’) OR ab:(‘**Botswana**’) OR ab:(‘**Burkina Faso**’) OR ab:(‘**Burundi**’) OR ab:(‘**Cabo Verde**’) OR ab:(‘**Cameroon**’) OR ab:(‘**Central African Republic**’) OR ab:(‘**Chad**’) OR ab:(‘**Comoros’)** OR ab:(‘Republic of Congo’) OR ab:(‘**Democratic Republic of the Congo**’) OR ab:(‘**Republic of the Cote d’Ivoire**’) OR ab:(‘**Djibouti**’) OR ab:(‘**Egypt**’) OR ab:(‘**Equatorial Guinea**’) OR ab:(‘**Eritrea**’) OR ab:(‘**Swaziland**’) OR ab:(‘**Ethiopia**’) OR ab:(‘**Gabon**’) OR ab:(‘**Gambia**’) OR ab:(‘**Ghana**’) OR ab:(‘**Guinea**’) OR ab:(‘**Guinea Bissau**’) OR ab:(‘**Kenya**’) OR ab:(‘**Lesotho**’) OR ab:(‘**Liberia**’) OR ab:(‘**Libya**’) OR ab:(‘**Madagascar**’) OR ab:(‘**Malawi**’) OR ab:(‘**Mali**’) OR ab:(‘**Mauritania**’) OR ab:(‘**Mauritius**’) OR ab:(‘**Morocco**’) OR ab:(‘**Mozambique**’) OR ab:(‘**Namibia**’) OR ab:(‘**Niger**’) OR ab:(‘**Nigeria**’) OR ab:(‘**Rwanda**’) OR ab:(‘**Sao Tome Principe**’) OR ab:(‘**Senegal**’) OR ab:(‘**Seychelles**’) OR ab:(‘**Sierra Leone**’) OR ab:(‘**Somalia**’) OR ab:(‘**South Africa**’) OR ab:(‘**South Sudan**’) OR ab:(‘**Sudan**’) OR ab:(‘**Tanzania**’) OR ab:(‘**Togo**’) OR ab:(‘**Tunisia**’) OR ab:(‘**Uganda**’) OR ab:(‘**Zambia**’) OR ab:(‘**Zimbabwe**’) OR ab:(‘**Eswatini’**)

**WEB OF SCIENCE**

(AB = Anthelmintic resistance OR AB=Endoparasite resistance OR AB=Antiparasitic resistance OR AB=nematode resistance OR AB=Benzimidazole resistance OR AB=imidazothiazole resistance OR AB=salicylanilide resistance OR AB=Tetrahydropyrimidine resistance OR AB=Macrocyclic lactones resistance) AND (AB=cattle OR AB=bovine OR AB=sheep OR AB=ovine OR AB=goat OR AB=caprine OR AB=chicken OR AB=poultry) AND (AB=Algeria OR AB=Angola OR AB=Benin OR AB=Botswana OR AB=Burkina Faso OR AB=Burundi OR AB=Cabo Verde OR AB=Cameroon OR AB=Central African Republic OR AB=Chad OR AB=Comoros OR AB=Republic of Congo OR AB=Democratic Republic of the Congo OR AB=Republic of the Cote d’Ivoire OR AB=Djibouti OR AB=Egypt OR AB=Equatorial Guinea OR AB=Eritrea OR AB=Swaziland OR AB=Ethiopia OR AB=Gabon OR AB=Gambia OR AB=Ghana OR AB=Guinea OR AB=Guinea Bissau OR AB=Kenya OR AB=Lesotho OR AB=Liberia OR AB=Libya OR AB=Madagascar OR AB=Malawi OR AB=Mali OR AB=Mauritania OR AB=Mauritius OR AB=Morocco OR AB=Mozambique OR AB=Namibia OR AB=Niger OR AB=Nigeria OR AB=Rwanda OR AB=Sao Tome Principe OR AB=Senegal OR AB=Seychelles OR AB=Sierra Leone OR AB=Somalia OR AB=South Africa OR AB=South Sudan OR AB=Sudan OR AB=Tanzania OR AB=Togo OR AB=Tunisia OR AB=Uganda OR AB=Zambia OR AB=Zimbabwe OR AB=Eswatini)

**MEDLINE**

1 Antiparasitic resistance.mp. 19

2 limit 1 to abstracts 17

3 antihelmintic resistance.mp. 0

4 limit 3 to abstracts 0

5 nematode resistance.mp. 530

6 limit 5 to abstracts 526

7 worm resistance.mp. 22

8 limit 7 to abstracts 21

9 Benzimidazoles resistance.mp. 9

10 limit 9 to abstracts 9

11 imidazothiazoles resistance.mp. 0

12 limit 11 to abstracts 0

13 salicylanilides resistance.mp. 1

14 limit 13 to abstracts 1

15 tetrahydropyrimidines resistance.mp. 0

16 limit 15 to abstracts 0

17 macrocyclic lactones resistance.mp. 2

18 limit 17 to abstracts 2

19 endoparasite resistance.mp. 8

20 limit 19 to abstracts 8

21 2 or 6 or 8 or 10 or 12 or 14 or 16 or 18 or 20 582

22 Cattle.mp. 408396

23 limit 22 to abstracts 314592

24 bovine.mp. 247318

25 limit 24 to abstracts 218744

26 pig.mp. 148491

27 limit 26 to abstracts 130342

28 swine.mp. 258718

29 limit 28 to abstracts 212613

30 sheep.mp. 159589

31 limit 30 to abstracts 120660

32 ovine.mp. 24028

33 limit 32 to abstracts 22197

34 goats.mp. 44048

35 limit 34 to abstracts 35202

36 caprine.mp. 4246

37 limit 36 to abstracts 3991

38 chicken.mp. 90141

39 limit 38 to abstracts 83280

40 poultry.mp. 70686

41 limit 40 to abstracts 56396

42 turkey.mp. 69916

43 limit 42 to abstracts 63757

44 23 or 25 or 27 or 29 or 31 or 33 or 35 or 37 or 39 or 41 or 43 945443

45 africa/ or africa, northern/ or algeria/ or egypt/ or libya/ or morocco/ or tunisia/ or "africa south of the sahara"/ or africa, central/ or cameroon/ or central african republic/ or chad/ or congo/ or "democratic republic of the congo"/ or equatorial guinea/ or gabon/ or "sao tome and principe"/ or africa, eastern/ or burundi/ or comoros/ or djibouti/ or eritrea/ or ethiopia/ or kenya/ or madagascar/ or rwanda/ or seychelles/ or somalia/ or south sudan/ or sudan/ or tanzania/ or uganda/ or africa, southern/ or angola/ or botswana/ or eswatini/ or lesotho/ or malawi/ or mozambique/ or namibia/ or south africa/ or zambia/ or zimbabwe/ or africa, western/ or benin/ or burkina faso/ or cabo verde/ or cote d'ivoire/ or gambia/ or ghana/ or guinea/ or guinea-bissau/ or liberia/ or mali/ or mauritania/ or niger/ or nigeria/ or senegal/ or sierra leone/ or togo/ 355697

46 limit 45 to abstracts 277081

47 swaziland.mp. 900

48 limit 47 to abstracts 767

49 46 or 48 277231

50 21 and 44 and 49 8

51 Anthelmintic resistance.mp. 1587

52 limit 51 to abstracts 1505

53 21 or 52 2064

54 44 and 49 and 53 51

Table s1: Quality assessment of included studies based on predefined methodological criteria. Each study was evaluated across seven domains: (i) clarity of study design, (ii) description of target animal species and sourcing, (iii) sample size consideration and power calculation, (iv) completeness of anthelmintic intervention details, (v) description and consistency of parasite identification and quantification methods, (vi) clarity of FECRT procedure and efficacy threshold, and (vii) differentiation of genera and/or species in FECR reporting. Scores were summed to provide a total, expressed as a percentage, and categorized as High, Moderate, or Low quality.

| Author | Is the study design clearly described, including number and definition of study groups and duration of the study? | Is the target animal species (including breed and age of the animals) and the sourcing of the animals clearly described in the study? | Does the study include a sample size consideration and power calculation based on a properly chosen experimental unit (animals or pens)? | Is the study description of the anthelmintic intervention complete, specifying the drug and formulation details and the treatment regimen (dosage and route)? | Is the method of parasite identification and quantification clearly described and is this consistent throughout the study? | Does the study clearly describe the FECR procedure and clearly indicate the minimum efficacy threshold? | Is the genera and/or species of the eggs present in the samples differentiated in FECR reporting? | **Total** | **Score %** | **Quality category** |
| --- | --- | --- | --- | --- | --- | --- | --- | --- | --- | --- |
| Bentounsi, et al. 2012 | 1 | 0.5 | 0 | 1 | 1 | 0 | 1 | **4.5** | **64.3** | **Moderate** |
| Atanasio et al 2002 | 0.5 | 0.5 | 0 | 1 | 1 | 1 | 0 | **4** | **57.1** | **Moderate** |
| Bersissa and Girma et al 2009 | 1 | 1 | 0 | 1 | 1 | 1 | 1 | **6** | **85.7** | **High** |
| Waruiru et al 1998 | 1 | 1 | 0 | 1 | 1 | 0.5 | 1 | **5.5** | **78.6** | **High** |
| Getachew et al 2016 | 1 | 1 | 0 | 1 | 1 | 1 | 0.5 | **5.5** | **78.6** | **High** |
| Boersema and Pandey et al 1997 | 1 | 1 | 0 | 1 | 1 | 0.5 | 1 | **5.5** | **78.6** | **High** |
| Mohammedsalih et al 2020 | 1 | 1 | 0 | 1 | 1 | 1 | 1 | **6** | **85.7** | **High** |
| Wondimu and Bayu et al 2022 | 1 | 1 | 0 | 1 | 1 | 1 | 0 | **5** | **71.4** | **Moderate** |
| Mphahlele et al 2021 | 1 | 1 | 0 | 1 | 1 | 1 | 0 | **5** | **71.4** | **Moderate** |
| Bakunzi et al 2008 | 0.5 | 0.5 | 0 | 1 | 1 | 1 | 0 | **4** | **57.1** | **Moderate** |
| Nsereko et al 2013 | 0.5 | 0.5 | 0 | 1 | 1 | 1 | 0 | **4** | **57.1** | **Moderate** |
| Byaruhanga et al 2013 | 0.5 | 0.5 | 0 | 1 | 1 | 1 | 0 | **4** | **57.1** | **Moderate** |
| Gabriel et al 2001 | 1 | 1 | 0 | 1 | 1 | 1 | 1 | **6** | **85.7** | **High** |
| Chaka and Gizaw et al 2009 | 0.5 | 0.5 | 0 | 0.5 | 0.5 | 0.5 | 0 | **2.5** | **35.7** | **Low** |
| Dreyer et al 2002 | 0.5 | 0.5 | 0 | 0.5 | 1 |  | 0 | **2.5** | **35.7** | **Low** |
| Mohammedsalih et al 2021 | 1 | 1 | 0 | 1 | 1 | 1 | 0 | **5** | **71.4** | **Moderate** |
| Nabukenya et al 2014 | 1 | 1 | 1 | 1 | 1 | 1 | 0 | **6** | **85.7** | **High** |
| Mukaratirwa et al 1997 | 0.5 | 0.5 | 0 | 1 | 0.5 | 0.5 | 0 | **3** | **42.9** | **Low** |
| Tsotesti et al 2013 | 0.5 | 0.5 | 0 | 1 | 1 | 1 | 0 | **4** | **57.1** | **Moderate** |
| Bentousi et al 2007 | 0.5 | 0.5 | 0 | 0.5 | 0.5 | 1 | 0 | **3** | **42.9** | **Low** |
| Molla et al 2023 | 0.5 | 0.5 | 0 | 0.5 | 0.5 | 1 | 0 | **3** | **42.9** | **Low** |
| Bakunzi et al 2003 | 0.5 | 0.5 | 0 | 1 | 0.5 | 1 | 0 | **3.5** | **50** | **Low** |
| Wanyangu et al 1996 | 0.5 | 0.5 | 0 | 0.5 | 0.5 | 0.5 | 0 | **2.5** | **35.7** | **Low** |
| Maingi et al 1998 | 0.5 | 0.5 | 0 | 1 | 1 | 1 | 0 | **4** | **57.1** | **Moderate** |
| Bentounsi et al 2006 | 0.5 | 0.5 | 0 | 0.5 | 0.5 | 1 | 0 | **3** | **42.9** | **Low** |
| Atanásio-Nhacumbe, et al 2017 | 0.5 | 0.5 | 0 | 1 | 0.5 | 1 | 0 | **3.5** | **50** | **Low** |
| Emsley et al 2023 | 0.5 | 0.5 | 0 | 1 | 1 | 1 | 0 | **4** | **57.1** | **Moderate** |
| Maurizio, et al 2024 | 0.5 | 0.5 | 0 | 0.5 | 1 | 0.5 | 0 | **3** | **42.9** | **Low** |
| **Mean Score** | **0.7** | **0.7** | **0** | **0.9** | **0.9** | **0.8** | **0.2** | **4.2** | **60** | **Moderate** |
